# Selective Photooxidation of Valencene and Thymol with Nano-TiO_2_ and O_2_ as Oxidant

**DOI:** 10.3390/molecules28093868

**Published:** 2023-05-04

**Authors:** Henry Martínez, Jane Neira, Álvaro A. Amaya, Edgar A. Páez-Mozo, Fernando Martínez Ortega

**Affiliations:** 1 Centro de Investigaciones en Catálisis, CICAT, Universidad Industrial de Santander, Piedecuesta 681011, Colombia; 2 Facultad de Ciencias Exactas, Naturales y Agropecuarias, Ciencias Básicas y Aplicadas Para la Sostenibilidad, CIBAS, Universidad de Santander, Bucaramanga 680003, Colombia

**Keywords:** photooxidation, TiO_2_ nanotubes, TiO_2_ nanoparticles, valencene, thymol

## Abstract

The selective photocatalytic oxidation with O_2_ as oxidant of valencene and thymol was evaluated using nanostructured TiO_2_ under UV-Vis radiation at atmospheric conditions. The effect of the morphology and optical properties of TiO_2_ nanotubes and aminate nanoparticles was studied. Different scavengers were used to detect the presence of positive holes (h^+^), electrons (e^−^), hydroxyl radicals (•OH), and the superoxide radical anion (O_2_^−^) during the photooxidation reaction. Superoxide anion radical is the main oxidizing specie formed, which is responsible for the selective formation of nootkatone and thymoquinone using aminated TiO_2_ nanoparticles under 400 nm radiation.

## 1. Introduction

The terpenes, such as valencene and thymol, present in essential oils obtained from aromatic plants [1,2,3] are responsible for their aromas and flavors [4]. Sesquiterpene valencene is a relatively inexpensive by-product of the processing of citrus products, used in the flavor and cosmetic fields thanks to its grapefruit odor and low perception threshold [1]. Thymol (2-isopropyl-5-methylphenol) is a monoterpene phenol [5,6] generally isolated from medicinal plants such as *Thymus vulgaris* and *Oroganum vulgare* [7,8]. It possesses antimicrobial, antifungal, and antioxidant properties and is a major component of the oils of thyme [9].

Nootkatone, a ketone derivative of valencene, is the most important and expensive grapefruit aroma. It is a sesquiterpenoid that decreases the somatic fat ratio, and its highly efficient production has been requested by the cosmetic, flavor, and fragrance industrial sectors. The conventional chemical process to produce nootkatone involves the oxidation of valencene using oxidants such as tert-butyl chromate, sodium dichromate, tert-butyl peracetate, tert-butyl hydroperoxide (TBHP), and molybdate ions as the catalysts [10]. Nootkatone has two isomers, (+)-nootkatone and (−)-nootkatone; the synthesis of enantiopure (+)-nootkatone was first reported by Yoshikoshi’s group from (−)-β-pinene, with an 11~14% overall yield by acid catalysis of cyclobutane cleavage–Aldol cyclization tandem reaction as a key step [11,12]. This last reaction was improved by Laine et al. via the stereoselective Grignard reaction, giving an overall yield of up to 33% [13]. However, the long and tedious work-up reaction procedure, the toxic solvent used, the strict reaction conditions, and the low overall yield make the total synthesis of (+)-nootkatone not the best choice from an economic and ecological point of view.

Alternatively, (+)-nootkatone can be prepared by the allylic oxidation of the (+)-valencene with tert-butyl chromate (carcinogenic) or sodium dichromate, as reported by Hunter et al. [14] and Shaffer et al., with 67% and 45%, respectively [1]. Tert-butyl peracetate was also used for allylic oxidation of valencene, obtaining nootkatone with 47% overall yield, but additional chromic acid was required for the oxidation of the nootkatol (intermediary) [15]. One-pot catalytic conversion of (+)-valencene with 75% yield into (+)-nootkatone with 99% selectivity has been realized with tert-butyl hydroperoxide in combination with silica-supported Co(OAc)_2_, Cu(OAc)_2_, or V(OAc)_2_ catalysts [16,17]. Nevertheless, the explosive, corrosive, and/or toxic properties of the systems mentioned above call for greener and more effective methods for preparing (+)-nootkatone from (+)-valencene. Recently, Guerra et al. [18] reported on the allylic oxidation of (+)-valencene with tert-butyl hydroperoxide using a copper–aluminum mixed oxide as a catalyst in the presence of L-proline with a yield to (+)-nootkatone of 40%. Using oxygen as an oxidant, valencene was oxidized with manganese porphyrin and obtained a conversion of 74%, with a selectivity of 38% to nootkatone and 21% to valencene-1,10-epoxide [19]. In addition, various biocatalysts, such as *G. pentaphyllum* cultures, green algae *Chlorella* species, fungi *Bothryosphaeria dothidea*, the lyophilisate of edible mushroom *Pleurotus sapidus*, and several bacterial cytochrome P450 enzymes, have also been studied for this transformation [20,21,22,23,24,25]. However, the costly culture conditions, the low conversion rate and yield, the inhibition of enzymes byproducts, and the presence of various byproducts still hamper the industrial preparation of (+)-nootkatone via biocatalysts. The discovery of new and more efficient enzymes in future research is necessary in order to diminish the cost of the biotransformation process.

Thymoquinone is an oxidation product of thymol with antitumor, hepatoprotective, and membrane lipid peroxidation inhibition activity [26]. Thymoquinone is only available in limited amounts from natural sources; therefore, its facile synthesis from cheap and abundant chemicals such as thymol has great demand [27]. Dockal et al. obtained thymoquinone by homogeneous oxidation of thymol and carvacrol in 71–84% and 79–93% yields using Co(II) (salen) catalysts in DMF, under oxygen flow at low pressure [28]. Thymoquinone was also efficiently obtained with 67–99% selectivity by the oxidation of thymol and carvacrol in acetonitrile in the presence of Mn(III) porphyrins and hydrogen peroxide as an oxidant [29]. Y zeolite-supported tetracationic Mn(III) porphyrin complex led to a conversion (<25%) of carvacrol with a conversion of thymol less at 18% after 24 h of reaction in acetonitrile, but showed a 100% selectivity towards thymoquinone [30]. Water soluble Mn(III) PEG-porphyrin is reported as a catalyst in a biphasic medium, water/hexane (1:1), for the oxidation of carvacrol and thymol into thymoquinone. The reactions were performed using TBHP as an oxidant in the presence of ammonium acetate as a co-catalyst, reaching 94% of the thymol conversion after 5 h of reaction. Catalytic transformation of the oregano essential oil revealed selective conversion of thymol and carvacrol into thymoquinone [31]. Gunay et al. reported the oxidation of thymol and carvacrol with KHSO_5_ catalyzed by iron phthalocyanine tetrasulfonate in a CH_3_OH-H_2_O mixture with 99% conversions of thymol in 1 h at room temperature. The major product of both oxidation reactions was thymoquinone and the formation of polymeric products as poly(2-isopropyl-5-methyl-1,4-phenylene oxide) and poly(5-isopropyl-2-methyl-1,3-phenylene oxide) was also mentioned [26].

Titania is one of the most widely studied materials to efficiently harvest solar light, which is one of the major challenges of our time. However, its overall efficiency for solar-driven applications is limited to the UV range due to its wide band gap (3.2 eV, for the anatase phase) [32,33,34]. Currently, the main approaches to improve its sunlight-driven photocatalytic efficiency are band gap narrowing and photosensitization. However, it is possible to tune the band gap by varying the shape, size and composition of the nanostructures. TiO_2_ nanomaterials have been prepared by partially reducing and by metal-ion implantation or nonmetal doping [35,36]. Along these lines, remarkable interest has been shown toward the so-called “black titanias”, dark colored TiO_2_ nanoparticles (TNPs) capable of absorbing a wide range of wavelengths. García-Martínez et al. proposed the preparation of black organotitanium by the in situ incorporation of p-phenylendiamine (PPD) in the framework of anatase nanoparticles. Incorporation of the PPD inside the framework of anatase drives a significant reduction in the band gap of TiO_2_ materials (from 3.2 eV to 2.7–2.9 eV), exhibiting a higher photocatalytic activity under both UV and visible light [32,33,34].

On the other hand, morphology also plays an important role in photocatalytic activity with TiO_2_. The synthesis of nanostructured TiO_2_ products such as nanotubes (TNTs), nanowires (TNWs) and nanofibers (TNFs) has garnered interest lately compared to conventional microstructures due to their high surface-to-volume ratio [37]. In particular, 1D Nanotubes or nanorods may allow for a much higher control of the chemical or physical behavior. By diminishing the dimensions to the nanoscale, not only does the specific surface area increase significantly, but the electronic properties may also change considerably (owing, for example, to quantum size effects, the strong contribution of surface reconstruction, or the surface curvature). These effects may also contribute to drastically improve the reaction/interaction between a device and the surrounding media, thereby making the system more effective (kinetics), or even allow for entirely novel reaction pathways [38]. The motion of charge carriers is restricted or confined to the other two dimensions, and hence, it is known as 2D confinement or radial confinement. Besides the high aspect ratios that result from their large length-to-diameter ratios, 1D TiO_2_ nanostructures are known to exhibit large specific areas, enhanced light absorption/scattering, interfacial charge transport properties, and charge separation efficiencies in order to improve the photocatalytic activity of the TiO_2_ nanostructures [39].

TiO_2_ allows the selective photooxidation of olefins and aldehydes [40,41] using different oxidants, and their catalytic activity is related to the TiO_2_ morphology [42,43,44]. The modification of the TiO_2_ surface with carboxyl and hydroxyl groups [45,46] has been used to increase the absorption of visible light and diminish the recombination of electron holes to achieve high photocatalytic efficiency [47,48,49].

**Scheme 1:**
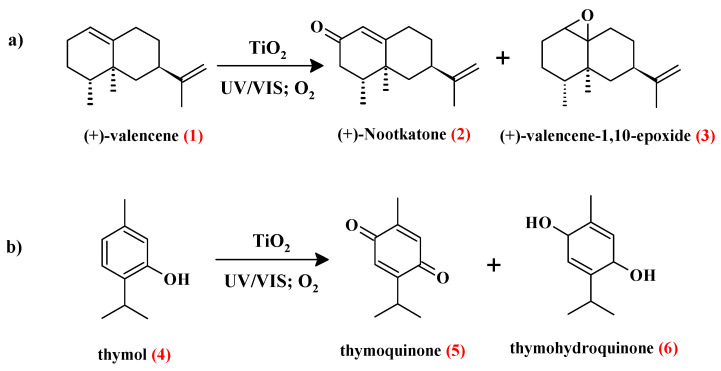
Chemical structure of the possible (**a**) valencene and (**b**) thymol selective photooxidation products.

In this work, the selective oxidation of valencene and thymol was evaluated with TiO_2_ nanotubes (TNTs), TiO_2_ nanoparticles (TNPs), or aminated TiO_2_ nanoparticles (*a*-TNPs) under UV (365 nm) and visible (400 nm) radiation, using O_2_ as an oxidant at atmospheric conditions, in agreement with Scheme 1 reactions. Valencene (1) oxidation provided, as the main product, Nootkatone (2) and valencence-1,10-epoxide. Thymoquinone (5) was produced in greater quantity and thymohydroquinone (6) was obtained as byproduct in thymol oxidation. The aim of this paper is to study the effect of the morphology in TiO_2_ samples—nanotubes and aminate nanoparticles—on the optical properties and the application in the selective oxidation reactions of renewable raw materials such as monoterpenes.

## 2. Results

### 2.1. Characterization of the TiO_2_ Supports

**Figure 1:**
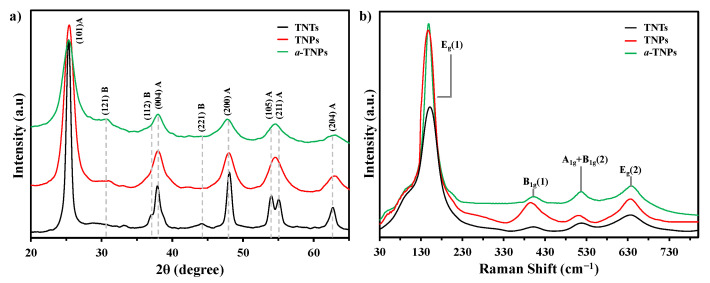
(**a**) XRD patterns and (**b**) Raman spectra of the TiO_2_ catalysts.

The wide-angle XRD pattern of the TNTs, TNPs, and *a*-TNPs showed diffraction peaks characteristic of anatase, as confirmed by the presence of the (101), (004), (200), (105), and (211) reflections (Figure 1a, JCPDS 21-1272) [50]. The 100% intensity peak (101) of anatase overlaps with the intensity peaks of 100% (120) and 80% (111) of the brookite phase for the nanoparticles [32,33,51]. (121), (112), and (221) reflections reveal the presence of the brookite phase (JCPDF #29-1360) [52]. Anatase–brookite mixture was reported previously in sol-gel and thermal treatments; these simultaneous phases are related with the temperature reaction. With sol-gel (90 °C) and hydrothermal (110 °C) methods, the number of collisions between particles increases, leading to a higher crystallization rate and resulting in the formation of the metastable polymorphs anatase or brookite [53]. Evidently, an increase in the temperature causes greater crystallization. García-Contreras et al., reported that the broad peaks in the anatase and brookite XRD patterns can be associated with the formation of nano-sized crystallites [54]. For photocatalytic applications, effective charge separation is a key factor. Among the three forms of TiO_2_, anatase is best known for its photocatalytic activity compared to rutile and brookite phases. However, increased charge separation can be achieved by the synergic effect of the mixed-phase composition. The mixture of the anatase and brookite phases has an effect on improving its activity and properties, such as with the Degussa P25 material [53]. Nachit et al. demonstrated that the presence of brookite phase in a TiO_2_-synthesized sample favors the reduction ability, leading to easier charge separation, and hence, to superior photocatalytic activity [55]. Raman spectra confirmed the presence of the anatase phase in the TiO_2_ supports (Figure 1b), since the characteristic peaks of the anatase phase were observed at 149 cm^−1^ Eg (1) mode, which corresponds to the symmetric stretching vibration of O-Ti-O, at 399 cm^−1^ mode B1g (1). Symmetric bending vibration of O-Ti-O, at 519 cm^−1^ modes (A1g + B1g doublet) were observed as well, corresponding to the antisymmetric bending vibration of O-Ti-O and at 638 cm^−1^ Eg mode (2) [51,56,57].

**Figure 2:**
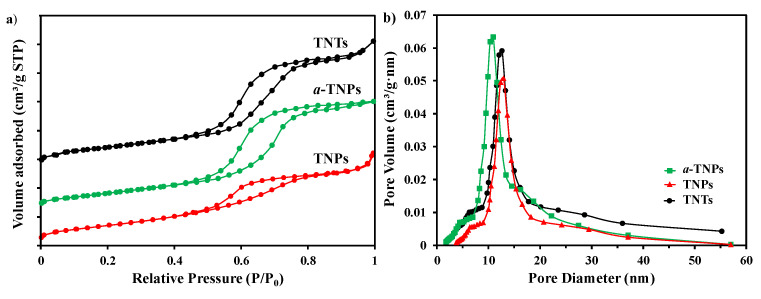
(**a**) N_2_ adsorption-desorption isotherms at 77 K for the different TiO_2_ prepared; (**b**) the corresponding pore size distributions obtained from the adsorption branch.

The BET analysis of the adsorption isotherm of N_2_ at 77 K allowed for the determination of textural properties for the TiO_2_ materials prepared, see Table 1. Figure 2a shows N_2_ adsorption-desorption isotherms. It is observed that all three samples—TNTs, TNPs, and *a*-TNPs—have type IV isotherms with large hysteresis loops that are characteristic of mesoporous solids, according to the IUPAC classification. A typical feature of Type IV isotherms is a final saturation plateau. The nearly horizontal plateau near the bulk saturation pressure indicates that all pores are completely filled with liquid-like adsorbate [58]. For TNTs and TNPs samples, the observed hysteresis extended to P/P_0_ ≈ 1 indicates the presence of large pores and condensation in inter-particle voids due to the fact that the adsorbed amount increases at higher relative pressures. In this case, capillary condensation is accompanied by a broad H2 hysteresis loop, indicating a wide distribution of pore sizes. This occurs when the pore width exceeds a certain critical width, which is dependent on the adsorption system and temperature; for nitrogen adsorption in cylindrical pores at 77 K, hysteresis starts to occur for pores wider than ~4 nm. The H2 hysteresis cycle corresponds to porous materials and an interconnected network of pores or ink-bottle neck shapes that are commonly found associated with compact non-uniform particles [59,60,61]. Figure 2b shows the corresponding pore size distribution curves of the samples calculated by the BJH method. TiO_2_ supports (TNTs, TNPs, *a*-TNPs) have broad pore size distribution with a maximum between 10–15 nm. The similarity of the textural parameters observed for both the control and the *a*-TNPs provides evidence that the incorporation of PPD in the anatase nanoparticles does not block the mesopores.

**Figure 3:**
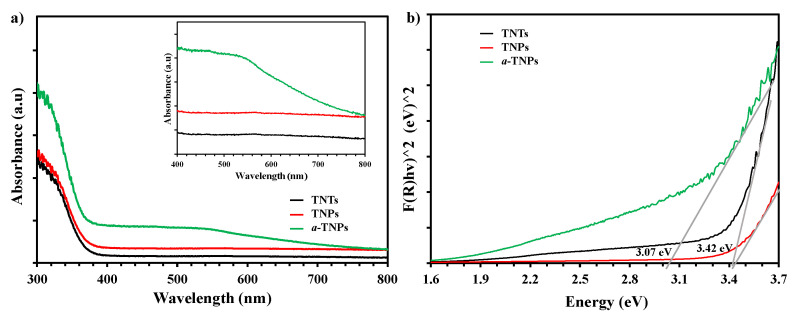
(**a**) UV-Vis absorption spectra and (**b**) Kubelka–Munk function vs. photon energy for the different TiO_2_ catalysts.

The Diffuse Reflectance UV-Vis Spectra (DRS) of the TiO_2_ samples are shown in Figure 3a. In this case, while TNPs and TNTs are only absorbed in the UV region (λ < 390 nm, band gap ~3.2 eV), the *a*-TNPs sample extends its absorption into the visible range (400–600 nm) due to its black coloration. All samples showed the typical band gap absorption in the UV region, corresponding to the electron transitions from the valence band to the conduction band (O2p → Ti3d). *a*-TNPs visible absorption can be due to the adsorption of nitrogen containing species on the surface of TiO_2_ nanoparticles that could result in a sub band gap of N2p between the O2p and Ti3d states [62].

To estimate the band gap of the TiO_2_ samples, the Diffuse Reflectance Spectroscopy (DRS) data were converted into the equivalent absorption coefficient using the Kubelka–Munk function [63]. The band gap calculations are based on the graph [F(R)hν]^2^ vs. the photon energy (Tauc graphs, inset), by finding the cut-off point with the x-axis of the line tangent to the curve, see Figure 3b [64]. The results observed in Figure 3b are according to the TiO_2_-PPD samples reported, where the Band gap values around 2.5 eV were obtained for amorphous/low crystalline, and the Eg value increased up to 2.7–2.8 eV for crystalline black organotitanias [32].

The aminated TiO_2_ nanoparticles sample presents a lower energy absorption band, around 3.07 eV (λ = 405 nm), due to indirect transitions of the anatase phase and PPD, which is markedly red-shifted with respect to the value of TNPs and TNTs, with energy for both around 3.42 eV (363 nm) [42,65]. In addition, the presence of the brookite phase has blue-shifted absorption values that are within the range of 3.3 to 3.48 eV with respect to the anatase phase [66]. Leyva-Porras et al. reported that 86–14% anatase–brookite mixture improves the thermal stability and effective charge separation, but with an increase in band gap energy value [53].

**Figure 4:**
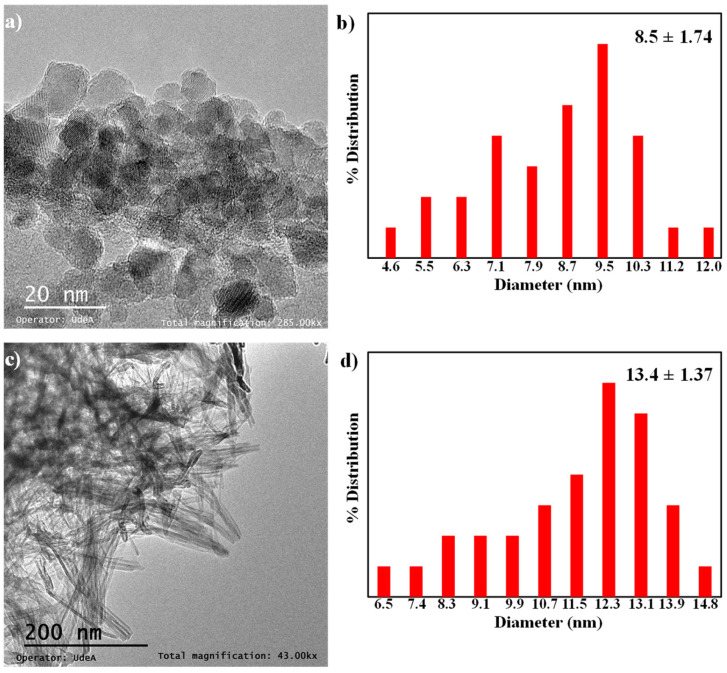
TEM micrographs and particle size distribution of (**a**) and (**b**) *a*-TNPs and (**c**) and (**d**) TNTs.

Figure 4 presents the TEM micrographs used to observe the morphology of the different nano-TiO_2_ catalysts. The external diameter of nanotubes and aminated TiO_2_ nanoparticles was determined with ImageJ software. In Figure 4a, it is observed that the diameters of the *a*-TNPs are in the range of 8.5 ± 1.74 nm. While TNTs are uniform tubular materials with an external diameter of around 13.4 ± 1.37 nm and lengths close to 500 nm, see Figure 4c. In both cases, the method of preparation used (alkaline hydrothermal treatment and a sol-gel) allows for the obtaining of nanoparticles of an approximately uniform size. SEM micrographs and EDS analysis of solids, as shown in Figure S4, provided evidence of the presence of nitrogen in *a*-TNPs and sodium in TNTs.

TiO_2_ thermogravimetric profiles are observed in Figure S1. A weight loss of less than 6% was observed at low temperatures (less than 120 °C), corresponding to physiosorbed water [57,67]. For the *a*-TNPs, a second weight loss of 16% was observed, corresponding to the decomposition of PPD at temperatures between 370 to 550 °C [32].

### 2.2. XPS Analysis

**Figure 5:**
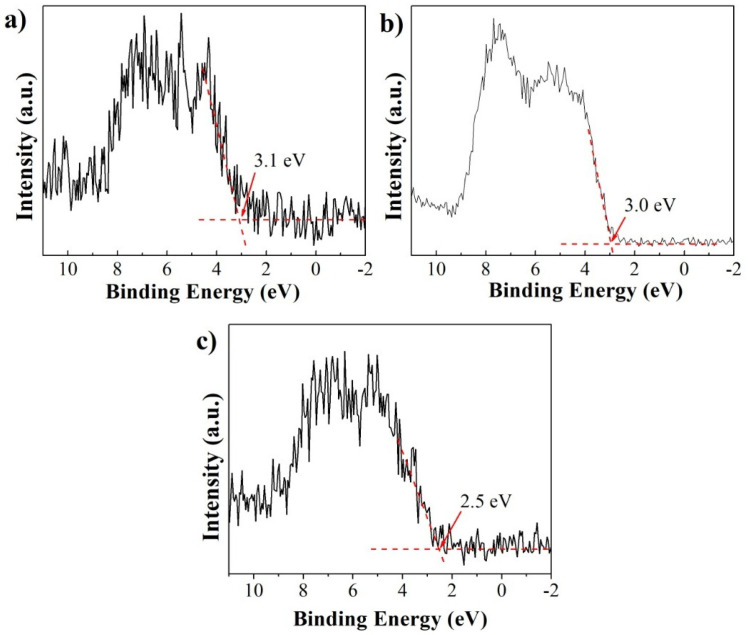
XPS valence band region for (**a**) TNPs, (**b**) TNTs and (**c**) *a*-TNPs.

XPS analysis was used to determine the surface chemical composition of the corresponding TiO_2_ catalysts. The global XPS spectrum is displayed in Figure S2, see Supplementary Information. Additionally, the XPS valence band region is used to determine the maximum of the VB for (a) TNPs, (b) TNTs, and (c) *a*-TNPs, as shown in Figure 5. The band gap of the TiO_2_ samples was calculated using the region between −2 to 11 eV, according to a graphical method [68]. The band gap of TNPs (3.1) and TNTs (3.0) are in agreement with the TiO_2_ materials reported [69]. The *a*-TNPs 2.5 eV band gap suggests that the amination process causes a reduction in the band gap with respect to TNPs associated with the incorporation of the PDD into the titania framework [32].

**Figure 6:**
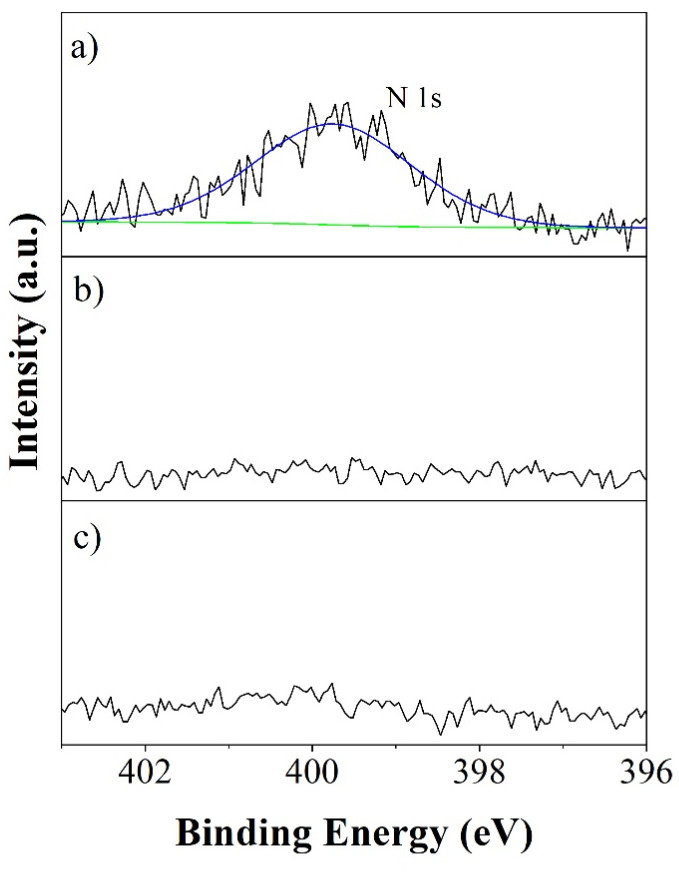
N 1s signal for (**a**) *a*-TNPs and (**b**) TNPs and (**c**) TNTs samples analyzed by XPS.

Figure 6 shows evidence of the presence of nitrogen on the surface of the *a*-TNPs sample. As expected, this signal was not observed on TNPs and TNTs samples, since that N was only included in the TiO_2_ sample from the PDD reagent used during their preparation. The binding energy for N 1s signal from the *a*-TNPs sample was about (399.8 eV). This value is expected for species where N-Ti-O bonds are present [70,71]. Besides, this binding energy corresponds to the chemical environment of the N atoms in the dopant agent used in this work (PPD), providing evidence that N from PPD was fixed in the TiO_2_ that was synthesized. The N/Ti ratio shown on the *a*-TNPs sample was about 0.095, i.e., 9.5 N atoms per 100 Ti atoms.

A Ti 2p3/2 XPS signal of 458.9 (TNPs), 458.8 (TNTs,) and 458.7 eV (*a*-TNPs) indicates the presence of Ti^+4^. Furthermore, additional lower binding energy components associated with the presence of reduced Ti^3+^ centers have not been detected by XPS after a careful deconvolution of the Ti2p peaks, which confirms the octahedral coordination of Ti^+4^ in aminated TiO_2_ nanoparticles (see Figure S3) [34,72,73]. Therefore, the modification of the band gap is likely caused by the incorporation of the PPD into the titania framework. The potential energy of the N 2p orbital is greater than the O 2p orbital, which raises the valence band of nitrogen above the valence band of oxygen, obtaining a narrower forbidden band [74].

**Figure 7:**
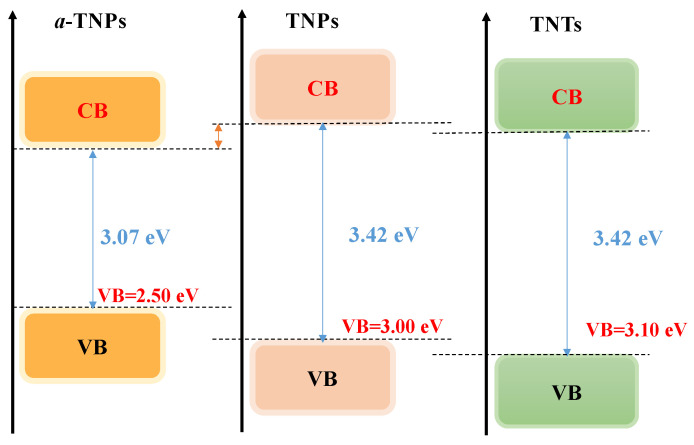
Band diagram for TiO_2_ catalysts, values calculated from DRUV and XPS measurements, for these solids.

Figure 7 displays the band diagram for the TiO_2_ samples prepared, calculated from DRS (Diffuse Reflectance Spectroscopy) and XPS measurements. The *a*-TNPs sample is the only sample that can absorb visible radiation, according to the band diagram, perhaps associated with the incorporation of PPD in the TiO_2_ structure. Previously, Hejazi et al. [62], reported that the significant increase in the magnitude of the photocurrent in the visible region can be related to formation of sub-band gaps with much lower values, i.e., with the Eg-value of ≈ 2.5 eV for the *a*-TNPs. Hence, this can be due to the adsorption of nitrogen-containing species on the TiO_2_ nanoparticles that could result in a sub-band gap of N2p between the O2p and Ti3d states [62]. J. García-Martínez et al., in their work with the preparation of visible-light-activated black organotitanias, concluded that the modification of the band gap is likely caused by the incorporation of the PPD into the titania framework. As expected, the band gap of the control titanias is 3.1–3.2 eV, independent of the synthesis conditions used to prepare them [32].

### 2.3. Valencene and Thymol Photooxidation

**Figure 8:**
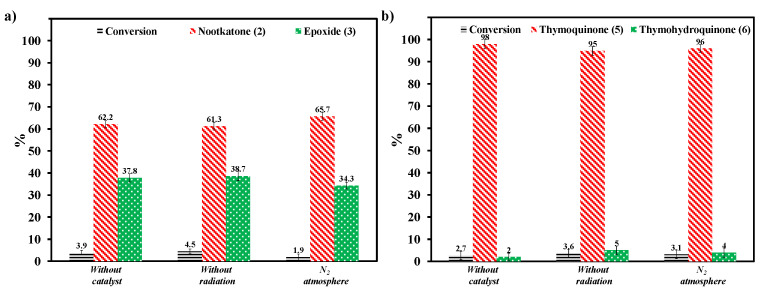
Selectivity and conversion for blank tests after 5 h of reaction of (**a**) valencene and (**b**) thymol.

The catalytic activity in the valencene and thymol oxidation with O_2_ using UV or Vis radiation was evaluated with TNTs, TNPs, and *a*-TNPs. The following blanks of the photooxidation of valencene and thymol were evaluated: (1) with radiation without catalyst, (2) with catalyst without radiation, and (3) in an N_2_ atmosphere. It is noted that in the absence of catalyst, radiation, and oxygen, the conversions are less than 5%, indicating that the reaction is photocatalytic, see Figure 8. However, the conversion was very low and perhaps associated with the sensitivity of the terpenes to light or the presence of radicals. The presence of radical oxygen species in the valencene solution may be responsible for the oxygenated products, which decrease in the N_2_ atmosphere. In the case of thymol, which is more photosensitive, the conversion and selectivity rise slightly under N_2_ atmosphere and irradiation.

**Figure 9:**
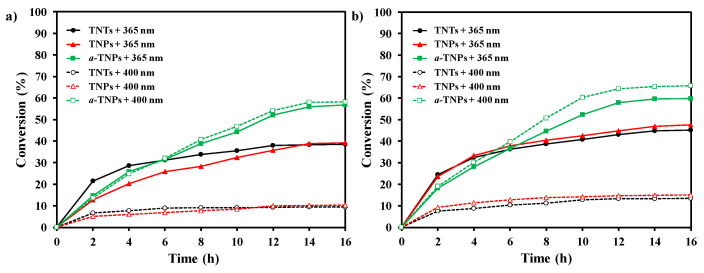
Conversion (%) on time of (**a**) valencene and (**b**) thymol using catalyst and UV or Vis radiation [Reaction conditions: 15 mg of nano-TiO_2_, substrate: 1 × 10^−2^ M; solvent: CH_3_CN, oxidant: O_2_, ambient pressure and temperature].

Figure 9 shows the conversion in the photooxidation of (a) valencene and (b) thymol with the three catalysts (TNPs, *a*-TNPs, and TNTs) at 25 °C using UV (365 nm) and Vis (400 nm) radiation. *a*-TNPs permit a higher conversion of valencene and thymol (above 50%) than TNTs and TNPs using UV and Vis radiation. Thymol photooxidation performed with a-TiO_2_ was more susceptible to Vis radiation than that performed with UV, where the presence of the PDD in the TiO_2_ helps the visible absorption. TiO_2_ nanoparticles and nanotubes without amination present a lower conversion (above 40%) with UV radiation, but the conversion does not exceed 12% when Vis radiation is used. The catalytic results agree with the absorption properties of the different TiO_2_ depending on the band gap calculated by DRS and XPS (Figure 7).

**Figure 10:**
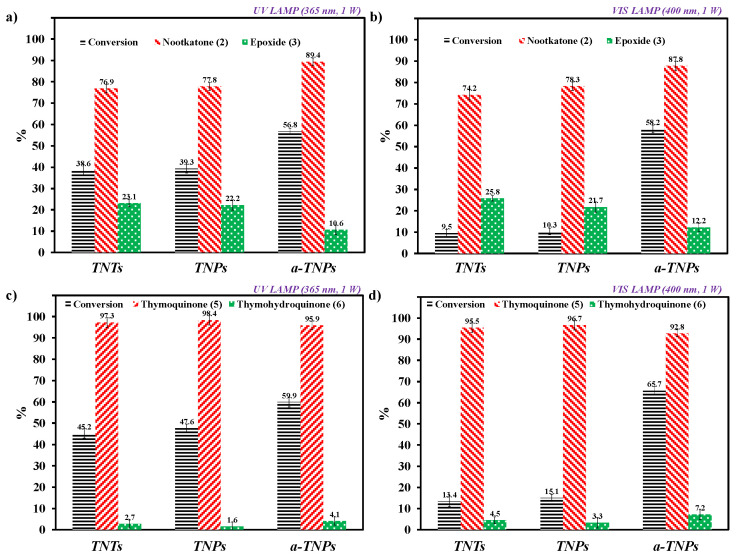
Conversion and selectivity in photooxidation of: (**a**) valencene with UV lamp (365 nm, 1 W), (**b**) valencene with VIS lamp (400 nm, 1 W), (**c**) thymol UV lamp (365 nm, 1 W) and (**d**) thymol with VIS lamp (400 nm, 1 W). [Reaction conditions: 15 mg of catalyst, substrate: 1 × 10^−2^ M; solvent: CH_3_CN, oxidant: O_2_, at ambient pressure and 25 °C].

Selectivity during the photooxidation of valencene and thymol does not change with UV or Vis radiation. Nootkatone and valencene-1,10-epoxide were obtained as products in valencene oxidation (Figure 10a,b). Thymoquinone and thymohydroquinone were obtained as products in thymol oxidation (Figure 10c,d). Nootkatone (>75%) and thymoquinone (>95%) are the main oxidation products, (with valencene epoxide and thymohydroquinone as byproducts). The oxidation products formed are due to photogenerated holes or by the attack of superoxide anions (O_2_^•−^) and •OH radicals depending on the solvent used [75,76]. C. Nunes de Melo et. al. proposed that superoxide anion (O_2_^•−^) is the responsible species for the formation of nootkatone and valencene epoxide employing manganese porphyrins as catalysts [19]. The formation of thymoquinone and thymohydroquinone may be due to the presence of the superoxide anion.

**Figure 11:**
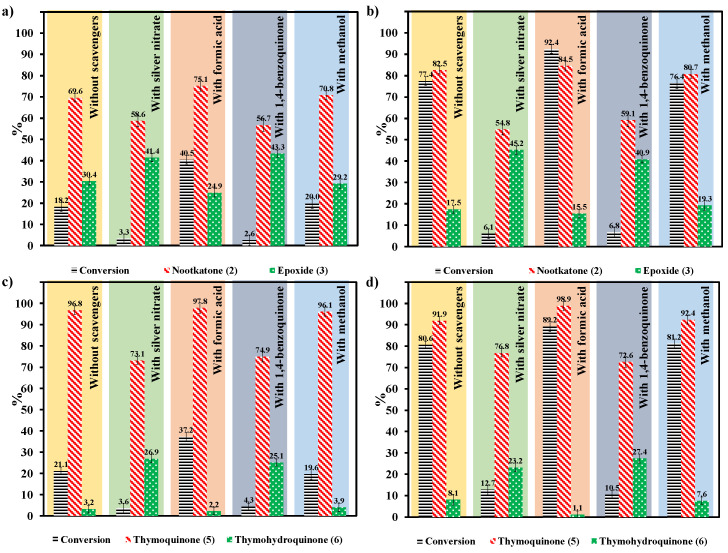
Conversion and selectivity in photooxidation of: (**a**) valencene with TNTs, (**b**) valencene with *a*-TNPs, (**c**) thymol with TNTs and (**d**) thymol with *a*-TNPs. [Reaction conditions: 15 mg of catalyst, substrate: 1 × 10^−2^ M; scavenger 1 × 10^−2^ M, VIS lamp (400 nm, 6 W), oxidant: O_2_, ambient pressure and temperature].

The catalytic role of the main active species in the photooxidation reaction was investigated using different scavengers: formic acid for holes (h^+^), silver nitrate for electrons (e^−^), methanol for •OH, and 1,4-benzoquinone (BQ) for O_2_^•−^ in the conversion and selectivity (Figure 11). The separation of electrons and holes is always recognized to be the initial step in the photocatalytic reaction mechanism [77,78]. The presence of silver nitrate drives a drop in conversion, since Ag^+^ capture produces electrons and diminishes the formation of superoxide (O_2_^•−^). When formic acid is used, the conversion is increased. When benzoquinone is used, the conversion drops below 10% due to the absence of O_2_^•−^.

**Scheme 2:**
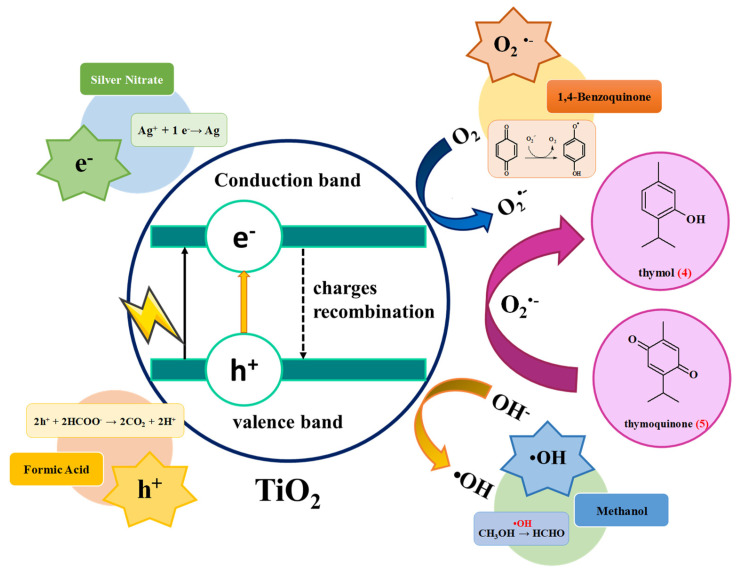
Scavengers reactions with TiO_2_ species: silver nitrate with electrons (e^−^), formic acid with holes (h^+^), 1,4-benzoquinone with superoxide radical anion (O_2_^•−^), and methanol with hydroxyl radicals (•OH).

Scheme 2 shows a proposed diagram for the formation of the oxidation products (nootkatone and thymoquinone) in the presence of scavengers, suggesting that the superoxide is the principal oxidant specie present. In a previous study, Goto et al. [79] quantitatively studied the reduction products obtained from molecular oxygen in titanium dioxide (TiO_2_)-photocatalyzed reactions. In photocatalysis, electrons react with molecular oxygen via the reductive pathway to generate O_2_^•−^. Additionally, O_2_^•−^ has been reported that suitable solvents for O_2_^•−^ generation are acetonitrile and DMSO.

## 3. Discussion

Olefinic terpenes constitute a promising source of abundant and cheap biorenewable raw material, whose catalytic transformation could generate economic benefits and various applications in the chemical industry. Among the many catalytic chemical processes used for the valorization of these agroresources, oxidation reactions are a key methodology for the valorization of some terpenes by obtaining oxyfunctionalized compounds, which have great application in the pharmaceutical, perfumery, and flavoring industries. The oxidation reaction is affected by the nature of the catalyst and the reaction conditions in the formation of an epoxide through the epoxidation of the double bond or allylic oxidation products by the functionalization of the C-H bond. Many catalysts have been evaluated in the oxidation of model terpenes using transition metal complexes or heterogeneous materials. In addition, organic peroxides (TBHP or peracetic acid) or green oxidants, such as hydrogen peroxide (H_2_O_2_) or molecular oxygen (O_2_), have been used as oxidants. The industry is guided by the use of heterogeneous catalysts instead of homogeneous ones. Currently, one of the main challenges in oxidation processes is selectivity control, despite achieving high catalytic conversions in allylic or epoxy products. This has motivated the development of research in the design of highly selective catalysts. In this sense, nanocatalysts have been proposed, due to their properties in terms of shape, structure, and morphologies, that provide new surface reactivities and could be suitable for a selectivity transformation of terpenes [80]. Previously, we reported the selective photooxidation of monoterpenes such as (R)-limonene, α-pinene, β-pinene, and camphene using the same TiO_2_ solid with different morphologies and textural properties. In these cases, TiO_2_ was used as support for dioxo-Mo complexes with different ligands [81,82,83]. However, the preparation of the catalyst requires several steps and the selectivity of product reactions is directed to monoterpene epoxides. The advantage with the catalytic system evaluated in this article is mainly the easy preparation of TiO_2_ catalysts and their evaluation in the allylic photooxidation of valencene and thymol.

The development of photocatalysts for the conversion of organic compounds selectively has been proposed with the aim of developing efficient, selective, environmentally friendly, and high-performance photocatalytic methodologies. Photocatalysis has currently advanced in the selective preparation of a wide variety of organic compounds, which industrially use complex preparation processes. The use of semiconductors in photochemistry, electrochemistry, inorganic, organic, physical, environmental, and polymer chemistry has been relevant. When a semiconductor surface is irradiated with photons of energy equal to or greater than the band gap of the semiconductor, an electron in the valence band is excited to the conduction band, thus creating a hole, h^+^, in the valence band. The photogenerated h^+^ and e^−^ species formed can oxidize and reduce other species through various mechanisms [84].

The properties of the anatase phase of TiO_2_, such as a lower rate of electron hole pair recombination and a higher adsorption capacity, have allowed its wide use in photocatalytic reactions in the selective oxidation of organic compounds. The efficiency of the reactions and the control of the selectivity can be increased by suitable modifications in the photocatalysts. Among the photocatalysts used in organic transformations, TiO_2_ has been widely used due to its environmentally friendly nature, low cost, and good catalytic properties. However, the overall efficiency of TiO_2_ for solar powered applications is limited to the UV range due to its bandwidth gap (3.2 eV, for the anatase phase). Currently, the main approaches to improve the sunlight photocatalytic efficiency of TiO_2_ are band gap narrowing and photosensitization. An alternative approach to improve the visible and infrared absorption of titania is based on the incorporation of dyes into the anatase framework during its synthesis through so-called “sol-gel coordination chemistry”. The in situ incorporation of p-phenylenediamine (PPD) within the framework of anatase nanoparticles was reported by J. García-Martínez et al., where the effective incorporation of PPD within the nanoparticles allows a significant reduction in the band gap of these materials (from 3.2 eV to 2.7–2.9 eV). This modification improved the photocatalytic efficiency of TiO_2_ with visible radiation [32].

Recently, Zhang et al. provided a review of updated information on the source and production of valencene and nootkatone, their physicochemical and bioactive properties, industrial applications, and safety and pharmacokinetics evaluation [10]. This study mentioned that Nootkatone, a ketone derivative of valencene, is a high economic value ingredient for the flavor and fragrance industry. In terms of commercial production, valencene and nootkatone are usually prepared by chemical synthesis, even though they are widely found in natural sources. The classical chemical process to produce nootkatone involves the oxidation of valencene using tert-butyl chromate, sodium dichromate, tert-butyl peracetate, TBHP, and molybdate ions as the catalysts. Additionally, a recent study found that Fe^2+^-chelates were used to convert (+)-valencene into (+)-nootkatone. Another study also showed a powerful strategy for the production of nootkatone from valencene using metalloporphyrins as a catalyst.

Furthermore, Bayraktar et al. mentioned that the oxidation of aromatic monoterpenes with hydrogen peroxide is a reaction applied at industrial level [85]. In their work, oregano essential oils rich in carvacrol (47.6%) and thymol (25.1%) were easily oxidized by hydrogen peroxide to oils containing thymoquinone (19.1–63.3%) as the main component in the presence of Fe(III) meso-tetraphenylporphyrin or Fe(III) phthalocyanines. The oxidation of carvacrol with hydrogen peroxide has also been studied using Mn(III) porphyrin complexes and Keggin-type tungstoborates. Oxidation of carvacrol by Keggin-type tungstoborates yielded a mixture of benzoquinones containing a small amount of thymoquinone, whereas for Mn(III) porphyrin complexes, oxidation of carvacrol selectively yielded thymoquinone. By using zeolite-encapsulated metal complexes, thymoquinone can be obtained through catalytic oxidation of carvacrol. Oxidation of carvacrol and thymol in the presence of Y-zeolite-entrapped Mn(III) tetra-(4-N-benzylpyridyl) porphyrin has been performed. The oxidation of carvacrol (<25% conversion) and thymol (<18% conversion) provided thymoquinone with 100% selectivity. However, leaching of the porphyrin complex from the zeolitic matrix occurred in the presence of H_2_O_2_ and was accompanied by a partial collapse and changes in the crystalline structure, causing irreversible deactivation.

Photocatalysis offers an alternative green route for the production of organic compounds. Photocatalytic reactions carried out in non-aqueous systems usually obtain rather high chemical yields of oxidation products, although sometimes with very low quantum yields/efficiencies. Both semiconductors and various organic species can be used as catalysts, with increasingly good results in terms of yields and selectivity. Heterogeneous metal oxide catalysts, however, are more easily recyclable, since they can be simply separated and usually are not readily deactivated, or, when deactivation occurs, it is a reversible process [86].

In their critical review about heterogeneous photocatalytic organic synthesis, Donia Friedmann et al. described that unlike the photocatalysts for organic degradation, the photocatalysts for organic synthesis should be highly customized on a case-by-case basis. Attention should be given to photocatalysts with the potential to be activated by the visible light spectrum in order to achieve cost effectiveness of the heterogeneous photocatalytic organic synthesis. The potential to utilize visible light for photocatalyst activation could mean even greater economical and environmental advantages. Additionally, in their review, they highlight that the application of heterogeneous semiconductor photocatalysis to organic synthesis presents more difficulties compared to applications such as the degradation of organic contaminants. All kinds of photocatalytic applications are based on the photoinduced charge transfers occurring on semiconductor interface with electrons and holes utilized as reductants and oxidants, respectively. The key issue in utilizing photocatalysis for selective organic synthesis is determining how to control the ways of interfacial charge transfer so that only the specific functional groups in substrate organic molecules are selectively transformed while the rest of the molecular structure remains intact. This review emphasizes that each photocatalyst needs to be optimized for a specific organic synthesis reaction on a case-by-case basis, since the selectivity control should depend on the molecular structure and property of the specific organic substrate as well as the photocatalyst properties [87]. Previously, we used the advantages of photocatalysis in different selective photooxidation reactions. Initially, TiO_2_ commercial (non-porous anatase) P25, mesoporous, and nanopowder with different textural properties were used. We provided evidence that larger specific surface areas with nanomaterials improve the photocatalytic results [57].

In our case, the selective oxidation of thymol and valencene, which are renewable raw materials obtained from essential oils of abundant aromatic plants in Colombia, were oxidized using aminated TiO_2_ nanoparticles. This catalytic system allows for the obtainment of oxygenated compounds utilizing O_2_ as an oxidant and UV-vis light.

The PPD incorporation into the titania (*a*-TNPs) decreases the band gap, allowing visible radiation absorption. The photocatalytic activity of *a*-TNPs is higher than TNTs and TNPs with visible light. The use of different scavengers indicates that the superoxide anion radical is the main oxidizing species formed, which is responsible for the selective formation of nootkatone and thymoquinone under 400 nm radiation [76].

This study provides evidence that selective photooxidation with O_2_ and visible light constitutes an environmentally friendly process that avoids the use of thermal energy and the presence of polluting byproducts [1,2,3,4,5,6,7,8,9,10]. The use of active semi-conductors in the visible region favors the formation of green oxidizing species (superoxide anion) obtained from O_2_. To achieve this, amine species can be introduced into the structure of the semiconductor to reduce the band gap, favoring the absorption of visible light.

In perspective, the catalytic system evaluated allows for the generation of oxy-functionalized terpenes, which can be the basis for the development of agroindustry specialized in the processing of natural extracts, rich in terpenes, into high-value products. This catalytic system is an environmentally friendly process, since it facilitates selective photooxidation by a nanostructured catalyst whose modification allows the absorption of visible light and the generation of oxidizing species using molecular oxygen (sustainable process). Additionally, the catalytic system proposed avoids the stoichiometric oxidants (highly polluting) and the thermal activation of molecular oxygen.

## 4. Materials and Methods

### 4.1. TiO_2_ Nanotubes Preparation (TNTs)

The preparation of Na_2_Ti_3_O_7_ nanotubes (sodium titanate nanotubes) was carried out by an alkaline hydrothermal treatment, using 1 g of commercial TiO_2_ nanopowder, which was added to a 10 M NaOH solution and stirred for 1 h. The mixture was transferred to a stainless-steel reactor and, during the first 6 h, was vigorously shaken inside the oven at 110 °C, until completion at 24 h. The precipitate was added slowly to a 0.1 M HNO_3_ solution for 12 h, it was washed with deionized water several times up to pH 7, and dried at 110 °C for 6 h. Finally, the precipitate was calcined at 400 °C for 2 h, and then the obtained TiO_2_ nanotubes powder was characterized by spectroscopic methods [51,52,53].

### 4.2. Aminated TiO_2_ Nanoparticles (a-TNPs)

The *a*-TNPs were prepared by a sol-gel methodology proposed by Jimenez et al. [32]; in this case, 80 mg of *p*-phenylenediamine (PPD) was dissolved in 35 mL of absolute ethanol at 40 °C, and stirred for 2 h. After, 5 g of titanium tetrabutoxide was added to aminated solution and it was left stirring at room temperature for 40 min. 60 mL of deionized water was added dropwise and it was kept stirring for 6 h. It was then placed in the oven at 90 °C for 24 h and the solid obtained was washed with acetone, filtered and left in the oven at 90 °C for 6 h.

### 4.3. Catalyst Characterization

TiO_2_ nanotubes and nanoparticles supports (TNTs and *a*-TNPs) were characterized by powder X-ray diffraction (XRD) using a Bruker AXS D8 Advance with monochromatized Cu Kα radiation (λ = 1.5418 Å) at 40 kV and 30 mA. The DRX pattern was recorded at 2θ value range (20–60°) with a step size of 0.01° and a step time of 0.4 s. TiO_2_ Raman spectra were performed using an integrated confocal Raman system (LabRAM HR Evolution HORIBA Scientific) spectrometer using a laser with an excitation wavelength at 532 nm, 10× objective and a power of 10 mW; ten accumulations of 2 s were used in each sample. Nitrogen adsorption-desorption isotherms at 77 K were obtained using a Micromeritics 3Flex apparatus. Before analysis, samples were degassed under a vacuum at 110 °C for 8 h. The specific surface area was determined from the linear part (0–0.23 P/P_0_) of the BET plot. The pore size distribution was determined by the BJH method applied to the adsorption branch. In addition, C, H, N, and S elemental analysis of the catalyst was carried out in an Elementar, Vario El Cube equipment. UV-Vis diffuse reflectance spectroscopy was used to determine band gap energy (Shimadzu UV 2401PC). X-Ray Photoelectron Spectroscopy (XPS) analysis was carried out in a SPECS^®^ XPS/ISS/UPS Surface Characterization Platform. The samples were analyzed using a monochromatic Al Kα X-ray source operated at 200 W/12 kV. The pass energy of the hemi- spherical analyzer was set at 60 eV for the high-resolution spectra. Charge compensation was performed using Flood Gun. The reference scale was calibrated by adjusting the carbon adventitious C–H to 284.8 eV. The Relative Sensitivity Factors (RSF) used for quantification procedures were: Mo 3d5/2 (5.73), Ti 2p (7.57), and S 2p (1.73). The pass energy of the hemispherical analyzer was set at 60 eV for high-resolution spectra and 100 eV for survey spectra. The XPS spectra were analyzed using CasaXPS software. All the signals were treated using static Shirley background and fitted using Gaussian–Lorentzian functions. The morphology of the TiO_2_ samples were done in the Transmission Electron Microscope (TEM) Tecnai F20 Super Twin TMP, field emission source, resolution of 0.1 nm at 200 Kv, maximum magnification at TEM 1.0 MX, GATAN US 1000XP-P camera. Samples for TEM studies were prepared by dipping a sonicated suspension of the sample in ethanol on a carbon-coated copper grid. The digital analysis of the TEM micrographs was performed using Gatam Digital MicrographTM 1.80.70 for GMS.

### 4.4. Catalytic Activity in the Photooxidation of Valencene and Thymol

10 mL of a terpene solution in acetonitrile (1 × 10^−2^ M) and 15 mg of catalyst were added to a 15 mL glass batch microreactor (ACEGLASS) equipped with a lamp holder, the temperature was maintained at 19 °C using a low atmospheric pressure. O_2_ was bubbled into the reaction medium under UV or Visible radiation for 9 h. Each experiment was carried out in duplicate, and during the progress of the reaction a sample was taken initially at 1 h and the following every 2 h. The samples were filtered and analyzed by gas chromatography (GC-HP-6890) using a Column HP-1 (100 m × 250 μm × 0.5 μm). The quantification of the products was carried out using toluene as an internal standard. The reaction products were identified by gas chromatography coupled to mass spectrometry (Agilent 5977B GC/MSD) of a single quadrupole and a HP-5ms capillary column (30 m × 0, 25 mm × 0.25 µm). We performed the photocatalytic experiment using some sacrificial agents to evidence the presence of active radicals and their participation during photocatalysis. In this experiment, silver nitrate, formic acid, methanol and *p*-benzoquinone (BQ) were used as electron (e^−^), hole (h^+^), hydroxyl radical (•OH) and superoxide radical (O_2_^•−^) scavengers, respectively.

## 5. Conclusions

TiO_2_ nanotubes and nanoparticles were successfully synthesized by a hydrothermal alkaline method and co-condensation of titanium tetrabutoxide with the *p*-phenylenediamine during the sol-gel synthesis, respectively. The PPD incorporation into the titania (*a*-TNPs) decreases the band gap, allowing the visible radiation absorption. The photocatalytic activity of *a*-TNPs is higher than TNTs and TNPs with visible light (400 nm). The different scavengers used indicate that the superoxide anion radical (O_2_^•−^) is the main oxidizing species formed, which is responsible for the selective formation of nootkatone and thymoquinone under 400 nm radiation by a allylic oxidation (C-H bond) of valencene and thymol.

## Figures and Tables

**Table 1:** Textural properties of TiO_2_ samples.

Sample	S_BET_ (m^2^·g^−1^)	V_p_ (cm^3^·g^−1^)	D_p_ (nm)
TNTs	289.81	0.60	13.1
TNPs	220.43	0.51	11.8
*a*-TNPs	188.73	0.62	13.6

## Data Availability

Not applicable.

## Supplementary Materials

The following supporting information can be downloaded at: https://www.mdpi.com/article/10.3390/molecules28093868/s1. Figure S1. Thermogravimetric profiles of nano-TiO_2_ catalysts. Figure S2. XPS spectra of the (a) TNTs, (b) TNPs and (c) *a*-TNPs. Figure S3. Ti 2p signal of the (a) TNTs, (b) TNPs and (c) *a*-TNPs. Figure S4. SEM micrographs and EDS analysis for (a) and (b) TNTs, and (c) and (d) *a*-TNPs.

## Sample Availability

Not available.
