# Supplementary material for: Selective Photooxidation of Valencene and Thymol with Nano-TiO2 and O2 as Oxidant
Source: Molecules. 2023 May 4;28(9):3868. doi: 10.3390/molecules28093868 (PMC10180157; doi:10.3390/molecules28093868)
Supplement: Supplementary file 1 [file molecules-28-03868-s001.zip › molecules-2265151-SI.pdf]

Selective Photooxidation of Valencene and Thymol with Nano-TiO_2 and O_2 as Oxidant

Henry Martínez [1], Jane Neira [1], Álvaro A. Amaya [2], Edgar A. Páez-Mozo [1] and Fernando Martínez Ortega [1,*]

[1] Centro de Investigaciones en Catálisis, CICAT, Universidad Industrial de Santander, Piedecuesta 681011, Colombia
[2] Facultad de Ciencias Exactas, Naturales y Agropecuarias, Ciencias Básicas y Aplicadas Para la Sostenibilidad, CIBAS, Universidad de Santander, Bucaramanga 680003, Colombia
* Correspondence: fmartine@uis.edu.co

SUPPORTING INFORMATION

Figure S1. Thermogravimetric profiles of nano-TiO_2 catalysts.

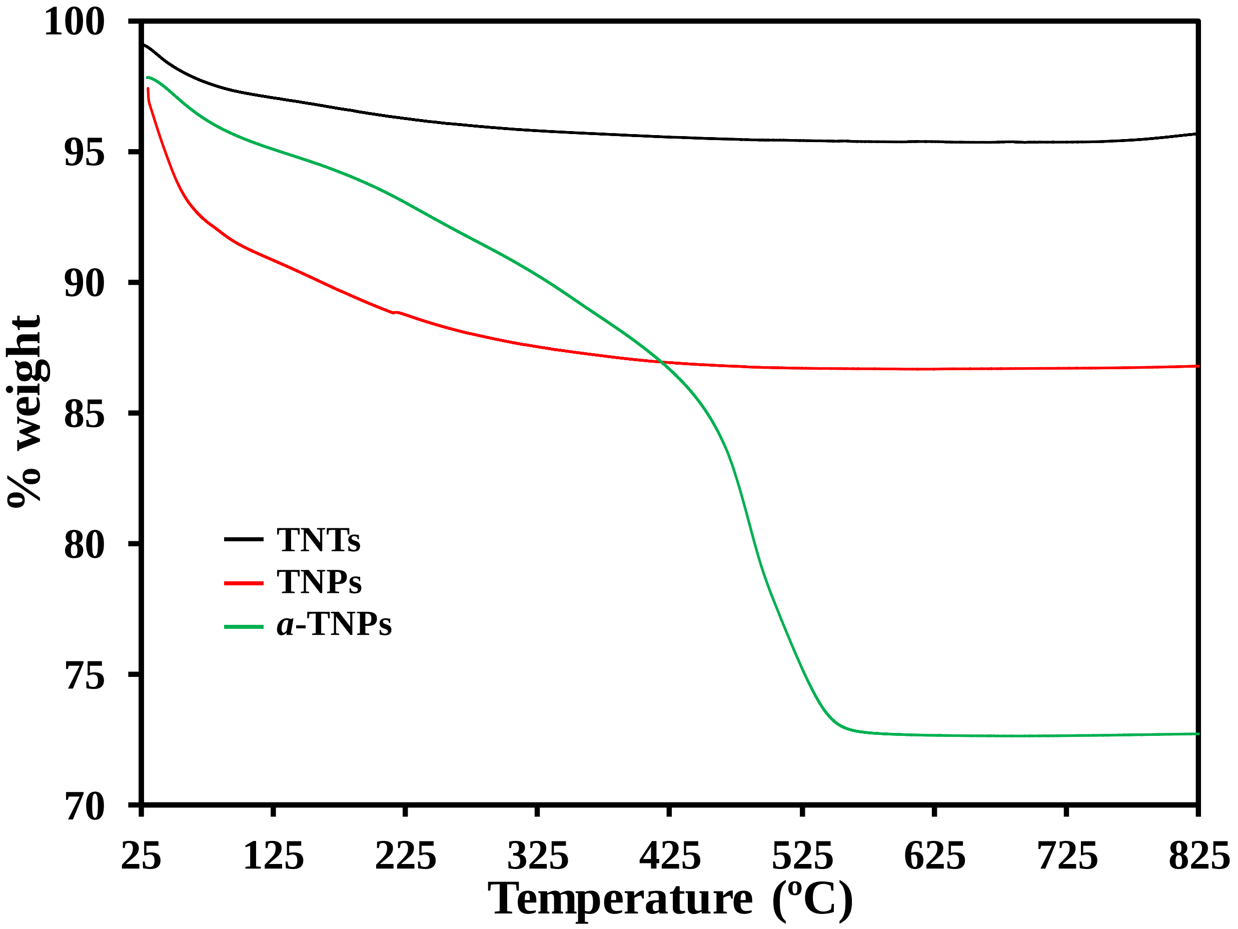

Figure S2. XPS spectra of the a) TNTs, b) TNPs and c) *a*-TNPs

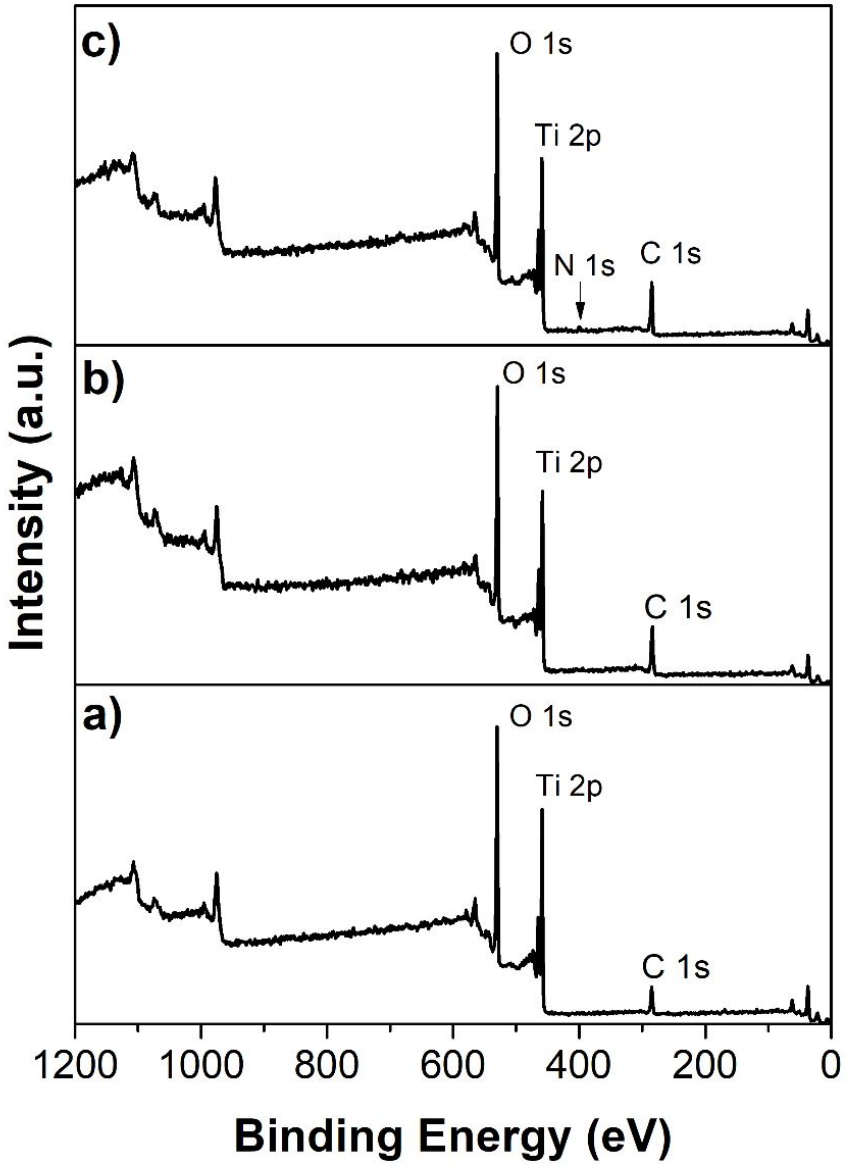

Figure S3. Ti 2p signal of the a) TNTs, b) TNPs and c) *a*-TNPs

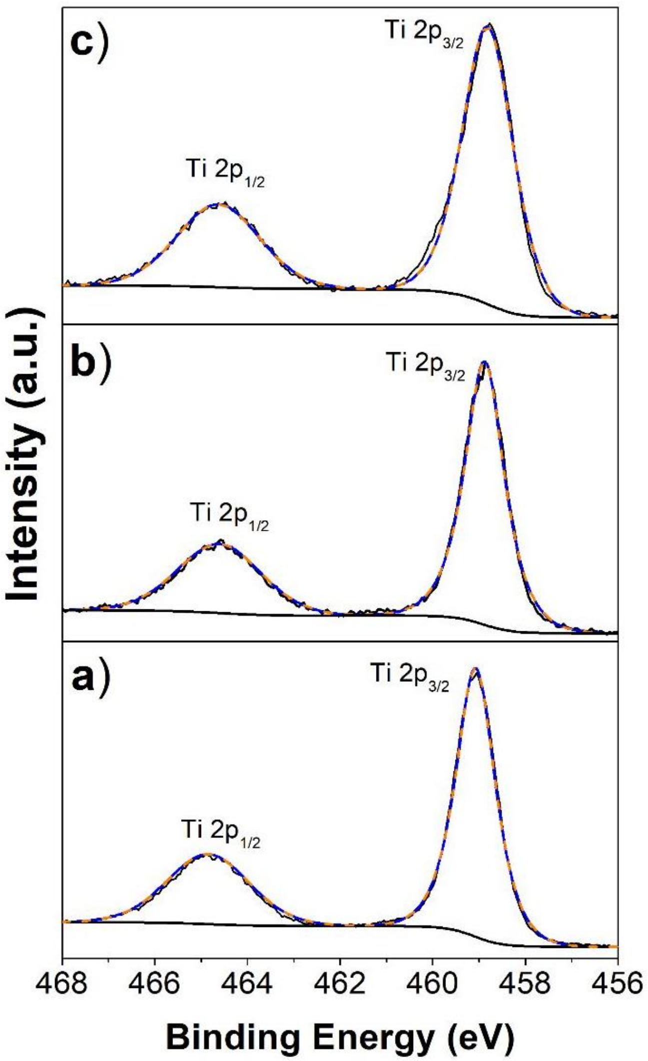

Figure S4. SEM micrographs and EDS analysis for a) and b) TNTs, and c) and d) *a*-TNPs.

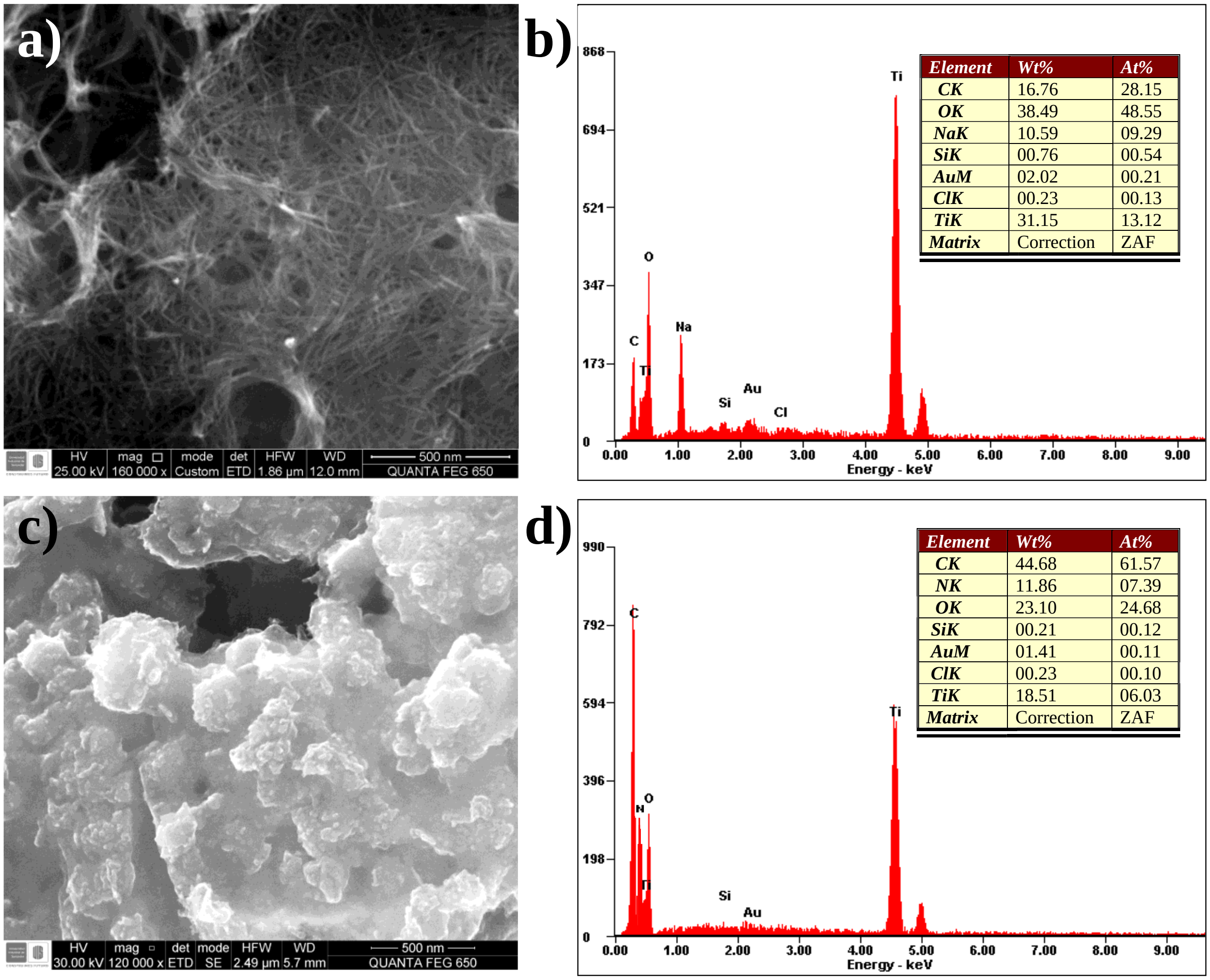

Element	Wt%	At%
CK	16.76	28.15
OK	38.49	48.55
NaK	10.59	09.29
SiK	00.76	00.54
AuM	02.02	00.21
ClK	00.23	00.13
TiK	31.15	13.12
Matrix	Correction	ZAF

Element	Wt%	At%
CK	44.68	61.57
NK	11.86	07.39
OK	23.10	24.68
SiK	00.21	00.12
AuM	01.41	00.11
ClK	00.23	00.10
TiK	18.51	06.03
Matrix	Correction	ZAF
